# Severe Hyperthyroidism Presenting with Acute ST Segment Elevation Myocardial Infarction

**DOI:** 10.1155/2015/901214

**Published:** 2015-07-14

**Authors:** Dayan Zhou, Zongjie Qu, Hao Wang, Zhe Wang, Qiang Xu

**Affiliations:** Department of Cardiology, Fifth People's Hospital of Chongqing, Renji Road No. 24, Nanan District, Chongqing 400062, China

## Abstract

*Introduction*. Acute myocardial infarction is life-threatening. A cardiac troponin rise accompanied by typical symptoms, ST elevation or depression is diagnostic of acute myocardial infarction. Here, we report an unusual case of a female who was admitted with chest pain. However, she did not present with a typical profile of an acute myocardial infarction patient. *Case Presentation*. A 66-year-old Han nationality female presented with chest pain. The electrocardiogram (ECG) revealed arched ST segment elevations and troponin was elevated. However, the coronary angiography showed a normal coronary arterial system. Thyroid function tests showed that this patient had severe hyperthyroidism. *Conclusion*. Our case highlights the possibility that hyperthyroidism may cause a large area of myocardium injury and ECG ST segment elevation. We suggest routine thyroid function testing in patients with chest pain.

## 1. Introduction

A patient is diagnosed with acute myocardial infarction if a typical rise and gradual fall (troponin) or a more rapid rise and fall (creatine kinase-MB, CK-MB) of biochemical markers of myocardial necrosis are seen with at least one of the following: (a) ischemic symptoms; (b) development of pathologic Q waves on the electrocardiogram (ECG); or (c) ECG changes indicative of ischemia (ST segment elevation or depression) [1]. When myocardial necrosis occurs, troponin rises before CK/CK-MB. Here, we report an unusual case of a 66-year-old female who was admitted with chest pain. The ECG revealed ST segment elevations and troponin was elevated. However, she did not present with a typical profile of an acute myocardial infarction patient.

## 2. Case Presentation

A 66-year-old Han nationality female, who had experienced a cerebral infarction 1 year previously and showed lingering muscle weakness in the right limbs, was admitted with chest pain and palpitations for 2 days. There was no significant family history of cardiac disease, and she had two healthy children. She did not have any risk factors, such as hypertension, diabetes, hyperlipidemia, or smoking.

Physical examination at the intensive care unit showed that her body weight was normal. Her temperature was 37.2°C, blood pressure was 105/65 mmHg, heart rate was about 131 beats per minute, and respiratory rate was about 24 breaths per minute. Heart sounds were normal. A small amount of rales could be heard at the bottom of both lungs.

**Figure 1:**
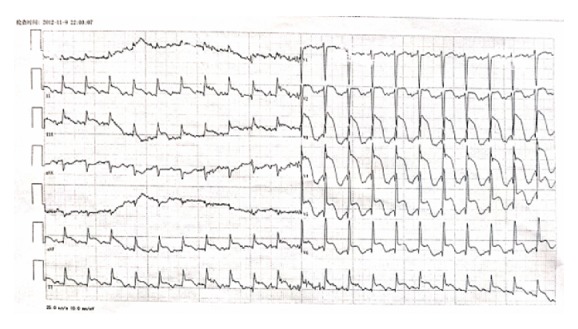
ECG upon arrival at hospital.

On admission, the ECG showed sinus tachycardia, 2 to 3 mm ST segment elevations in II, III, and aVF, and 2 to 9 mm ST segment elevations in V2 to V6 (Figure 1). A diagnosis of acute myocardial infarction was made, and the patient was immediately started on standard medication (aspirin, clopidogrel, atorvastatin, low-molecular-weight heparin, and metoprolol). An emergency coronary angiogram was not arranged; this is done only when patients have had chest pain within 12 hours. However this patient had chest pain for 2 days.

Laboratory workup revealed the following results: troponin I levels were markedly raised (7.959 *μ*g/L); myocardial enzymes (CK 299.0 IU/L, CK-MB 26.6 IU/L) and NT pro-Brain Natriuretic Peptide (NT-proBNP, 18497.0 pg/mL) were elevated; blood gas, glucose, liver function, and renal function were in normal ranges; initial laboratory tests revealed normal electrolytes; and plasma lipids showed surprisingly low concentrations (total cholesterol 2.8 mmol/L, triglycerides 0.83 mmol/L, LDL-cholesterol 1.75 mmol/L, and HDL-cholesterol 0.87 mmol/L).

However, the patient had persistent tachycardia (about 110–120 beats/minute) after drug treatment. Therefore, thyroid function tests were requested; these revealed hyperthyroidism (free T3 48.71 pmol/L (reference range 2.8–7.1), T3 8.59 nmol/L (1.3–3.1), free T4 > 100 pmol/L (12–22), T4 > 320 nmol/L (66–181), and thyroid-stimulating hormone < 0.005 *μ*IU/mL (0.27–4.2)). An acute myocardial infarction complicated by hyperthyroidism and threatened hyperthyroidism crisis could not be ruled out. She was therefore referred for an endocrine consultation and started on propylthiouracil.

**Figure 2:**
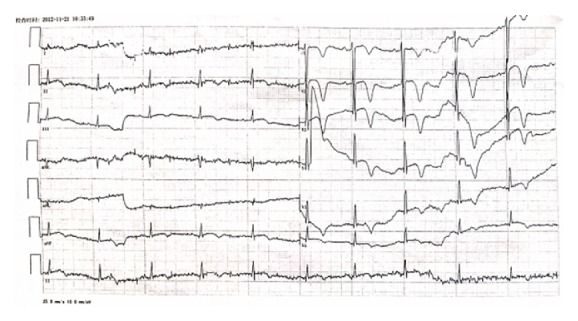
ECG 11 days after admission.

After 11 days of such a complicated condition, the patient's condition had stabilized. Blood pressure was 115/65 mm Hg and the heart rate was 95 beats per minute. ECG showed sinus rhythm and negative T waves on anterior and inferior derivations and no abnormal Q waves over the anterior and inferior leads (Figure 2). Echocardiography one week after admission showed normal left ventricle systolic function with the left ventricular ejection fraction of 65%. A thyroid ultrasound scan showed no focal lesions. Percutaneous angiography was also performed. Coronary angiography showed a normal coronary arterial system, a normal supply of blood to the heart, and no blockages.

**Figure 3:**
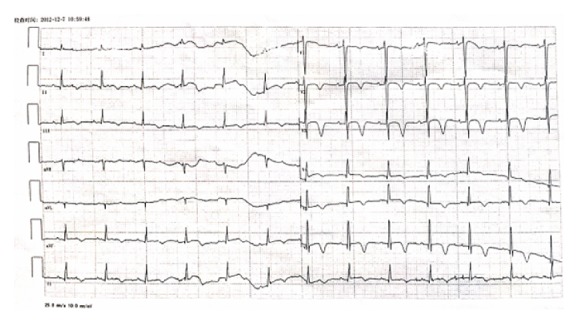
ECG 2 weeks after discharge from hospital.

The patient was successfully discharged after 2 weeks of treatment. A follow-up ECG showed no pathological Q waves over the anterior and inferior leads (Figure 3). She remained euthyroid on propylthiouracil, which was discontinued after 18 months. She denied any anginal symptoms.

Patient's ECG upon arrival at the hospital shows the following: sinus tachycardia, 2 to 3 mm ST segment elevations in II, III, and aVF, and 2 to 9 mm ST segment elevations in V2 to V6.

Patient's ECG 11 days after admission shows the following: sinus rhythm, T waves inversion on anterior and inferior leads, and no Q waves on anterior and inferior leads.

Patient's ECG 2 weeks after discharge from hospital shows the following: sinus rhythm, negative T waves on anterior and inferior derivations, and no Q waves.

## 3. Discussion

Myocardial infarction can occur with thyrotoxicosis [2, 3]. There is evidence that thyrotoxicosis is directly associated with the presence of a prothrombotic state [4]. Some long-term follow-up studies have revealed increased mortality from cardiovascular and cerebrovascular disease in persons with a history of overt hyperthyroidism [5], as well as in those with subclinical hyperthyroidism [6]. In another study, an elevated FT3 concentration was associated with a 2.6-fold greater likelihood of coronary events [7]. In the absence of fixed coronary artery disease or coronary artery spasm, thyrotoxicosis is rarely associated with acute myocardial infarction. Therefore, acute myocardial infarction and hyperthyroidism (especially thyrotoxic storm) are a difficult and dangerous combination. Although acute myocardial infarction and hyperthyroidism are curable when diagnosed early, they can be fatal if left undiagnosed or treated incorrectly.

The cause of myocardial ischemia and infarction in thyrotoxic patients with normal coronary arteries is unclear. It may be a result of in situ coronary thrombosis or a direct metabolic effect of thyroid hormone on the myocardium, or it may be secondary to supraventricular tachycardia or atrial fibrillation [8]. In addition, coronary vasospasm might be another factor contributing to the development of acute myocardial infarction. There are some reports of severe coronary artery spasm leading to myocardial infarction in young subjects with thyrotoxicosis, but without classical cardiovascular disease risk factors [3, 8, 9].

According to the typical ST segment elevation on the ECG, the chest pain, and the elevated myocardial enzymology, there was no doubt about the initial diagnosis of acute myocardial infarction in our patient. However, coronary angiography showed a normal coronary arterial system without any stenotic lesions. Admission ECG showed 2 to 3 mm ST segment elevations in II, III, and aVF and 2 to 9 mm ST segment elevations in V2 to V6, while long-term follow-up ECG showed no abnormal Q waves over the anterior and inferior leads. Therefore, the diagnosis of acute myocardial infarction had to be reconsidered. A thrombophilic tendency or severe spasm may be considered in our patient. According to the Third Universal Definition of Myocardial Infarction, our patient may have a type 2 myocardial infarction [10]. But we did not perform ergonovine or acetylcholine provocation test during coronary angiography. This was a limitation which could not differentiate vasospasm from causal disease. Thyroid function tests showed that this patient had severe hyperthyroidism, causing a large area of injury to the myocardium and leading to decreased cardiac function. Since it is known that thyroid hormones increase the demand for oxygen, the rapid elevation of oxygen utilization caused by thyrotoxicosis is likely responsible for this patient's myocardium injury. At the first physical examination rale sounds were heard and NT-proBNP levels were clearly elevated, indicating reduced left ventricular function. However, ventriculography or echocardiography was not performed in the acute phase. Transient systolic dysfunction of left ventricle, ST elevation, elevated troponin T and inverted T wave with no Q wave seem to be consistent with Takotsubo cardiomyopathy. Takotsubo cardiomyopathy is characterized by transient left ventricular dysfunction with chest symptoms, elevated cardiac enzymes, and ECG changes such as ST segment elevation and/or T-wave inversion without coronary artery occlusion [11]. The association of Takotsubo syndrome and hyperthyroidism has been reported before [12–14]. Therefore Takotsubo cardiomyopathy may be considered in our patient. However, such typical ST segment elevation in a patient with severe hyperthyroidism is rare and easy to misdiagnose.

## 4. Conclusion

Our case highlights the possibility that hyperthyroidism may cause a large area of myocardium injury and ECG ST segment elevation. We suggest routine thyroid function testing in patients with chest pain.
